# To PLEX or Not to PLEX for Amiodarone-Induced Thyrotoxicosis

**DOI:** 10.1155/2023/1563732

**Published:** 2023-11-20

**Authors:** Tania Ahuja, Olivia Nuti, Cameron Kemal, Darren Kang, Eugene Yuriditsky, James M. Horowitz, Raymond A. Pashun

**Affiliations:** ^1^Department of Medicine, The Leon H. Charney Division of Cardiology, New York University Grossman School of Medicine, USA; ^2^Department of Pharmacy, New York University Langone Health, New York, New York 10016, USA; ^3^Department of Advanced Practice Practitioners, New York University Langone Health, New York, New York 10016, USA

## Abstract

Amiodarone-induced thyrotoxicosis (AIT) carries significant cardiovascular morbidity. There are two types of AIT with treatment including antithyroid medications and corticosteroids and treatment of ventricular arrhythmias. Therapeutic plasma exchange (TPE) also known as “PLEX” may help remove thyroid hormones and amiodarone. We report a case of PLEX in an attempt to treat cardiogenic shock secondary to AIT. This case highlights the robust rapidly deleterious demise of AIT, specifically in patients with decompensated heart failure. The decision to PLEX or not to PLEX for AIT should be individualized, prior to definitive therapy.

## 1. Learning Objectives

- To describe the two distinct mechanisms and cardiovascular morbidity of amiodarone-induced thyrotoxicosis (AIT)
- To understand the unique use of therapeutic plasma exchange (PLEX) to mitigate the acute toxicity seen with AIT
- To review pharmacologic and nonpharmacologic attempts to remove thyroid hormones secondary to AIT

## 2. Introduction

Amiodarone, a class III antiarrhythmic, is widely used for the treatment of supraventricular and ventricular arrhythmias, paroxysmal atrial fibrillation (AF), and maintenance of sinus rhythm after cardioversion [1, 2]. Although rare, thyroid storm, a severe form of thyrotoxicosis, occurs in 3-10% of people treated with amiodarone, with increased incidence in geographical locations that remain iodine-depleted [3, 4]. The risk of amiodarone-induced thyrotoxicosis (AIT) increases with higher doses and lifetime exposure. One 200 mg tablet of amiodarone contains around 75 mg of iodine, with 10%, or 7 mg, released as free iodide daily. This is 40-50-fold above the daily recommended iodine intake [3, 5]. There are two distinct mechanisms of AIT, both of which can be refractory to standard therapies for thyrotoxicosis and result in cardiovascular collapse [6–8]. Type 1 AIT, also known as the Jod-Basedow phenomenon, typically occurs in the setting of underlying abnormal thyroid dysfunction as seen in Graves' disease or with autonomous nodular goiter. Here, the iodine from amiodarone causes an overproduction of thyroid hormones [3]. In contrast, type 2 AIT, also referred to as the Wolff-Chaikoff effect, is a thyroid destructive process, where iodine from amiodarone transiently inhibits organification, or the incorporation of iodine into thyroglobulin. This inhibits thyroxine (T4) and triiodothyronine (T3) production and release from the follicle within the first few days of amiodarone treatment [3, 5]. In addition, amiodarone inhibits type 1 5′-deiodinase activity in the periphery, limiting conversion of T4 to T3 [3]. Lastly, amiodarone has a direct toxic effect on thyroid cells, causing release of preformed T3 and T4 into circulation [3]. In the most severe form of AIT, patients may present with acute thyrotoxicosis, cardiac decompensation, and arrhythmias ranging from sinus tachycardia to atrial fibrillation or ventricular tachycardia [3]. Still, AIT poses diagnostic and therapeutic challenges, especially in those with advanced cardiovascular disease [8–10]. Treatment for type 1 AIT includes thionamides such as propylthiouracil (PTU) or methimazole (MMI), whereas treatment for type 2 AIT includes glucocorticoids [3, 9, 10]. Other therapies include medications to decrease the adrenergic response, such as beta-blockers or antiarrhythmics. The use of adjunctive therapies such as therapeutic plasma exchange (TPE) or plasmapheresis, also known as “PLEX,” may be considered in patients presenting with AIT with cardiovascular collapse or multiorgan failure, where definitive therapy including antithyroid medications and/or thyroidectomy are contraindicated. We report here the case of a patient that presented with AIT, with contraindications to thionamides and surgery, in whom TPE was attempted, as a bridge to thyroidectomy, but ultimately, refractory shock led to death.

## 3. Case Report

We report the case of a 52-year-old male with nonischemic cardiomyopathy and heart failure with reduced ejection fraction (HFrEF) of 35% with a primary prevention implantable cardioverter-defibrillator (ICD), atrial fibrillation, end stage renal disease on hemodialysis, hyperlipidemia, and hypertension presenting with an acute exacerbation of dyspnea. He reported subacute worsening shortness of breath and palpitations and was found to be in decompensated heart failure with cardiogenic shock. He was at dialysis earlier in the day prior to presentation to the hospital, where the session was ended early due to palpitations. Medications prior to admission included allopurinol 200 mg once daily, amiodarone 200 mg once daily, atorvastatin 20 mg once daily, carvedilol 12.5 mg twice daily, colchicine 0.6 mg daily, apixaban 5 mg twice daily, losartan 25 mg once daily, sevelamer 1600 mg three times daily, torsemide 40 mg once daily, trazodone 50 mg once nightly, and multivitamins. He stated that he was adherent to his medications, including amiodarone with no recent dose changes, and was under evaluation for renal and heart transplant, though has been experiencing worsening exercise tolerance over the past week, as well as worsening orthopnea and paroxysmal nocturnal dyspnea (PND). On arrival to the emergency room, he was normotensive at 129/81 mmHg, with a heart rate of 130 beats per minutes in atrial fibrillation, and his oxygen saturation was 98% on ambient air. Electrocardiogram (ECG) was notable for atrial fibrillation with rapid ventricular response at a rate of 123 beats per minute (bpm) in addition to right axis deviation and nonspecific ST segment and T wave changes. Laboratory data revealed a normal pH, lactate of 4.7 mmol/L which rapidly rose to 7.9 mmol/L, high-sensitivity troponin I of 130 ng/L (reference range < 36 ng/L), B-type natriuretic peptide of 1768 pg/mL (reference range < 100 pg/mL), an undetectable thyroid-stimulating hormone (TSH) (<0.02 mIU/L) (reference range 0.4-4.1 mIU/L), a free thyroxine level (T4) of 3.1 ng/dL (reference range 0.7-1.5 ng/dL), a free triiodothyronine (T3) of 2.6 pg/mL (reference range 1.71-3.71 pg/mL), an undetectable thyroid peroxidase antibody (TPO) of <3 IU/mL (reference range 0-5.5 IU/mL), an antithyroglobulin (TG) of 4.8 IU/mL (reference range 0-5 IU/mL), and a thyroid-stimulating immunoglobulin (TSI) of <0.1 (reference range < 0.54 IU/mL). Thyroid function tests obtained two years prior to starting amiodarone were within normal limits with a TSH of 2.23 mIU/L, a free T4 of 1.76 ng/dL, and a free T3 of 3.18 pg/mL (Table 1). Chest X-ray (CXR) was notable for cardiomegaly and bilateral hazy opacities concerning for pulmonary edema. Upon physical exam, he was noted to have significant exophthalmos with lid lag, tachycardia, diffuse bilateral crackles, diffuse abdominal tenderness, and cool lower extremities. He was admitted to the cardiac intensive care unit (CICU) for placement of a pulmonary artery catheter (PAC) to obtain hemodynamics and assess volume status to determine etiology of shock. Initial hemodynamics revealed a pulmonary artery pressure of 59/24 mmHg, central venous pressure of 18 mmHg, a cardiac index of 1.02 L/min/m^2^, and a systemic vascular resistance (SVR) of 3253 dyne^∗^sec/cm^5^. Electrical cardioversion was attempted for symptomatic atrial fibrillation with heart rates up to 160 beats per minute. A transthoracic echocardiogram (TTE) was completed demonstrating right ventricle hypokinesis, reduction in ejection fraction to 25% (previously 35% at 1 month prior to presentation), severe tricuspid regurgitation, and moderate-severe mitral regurgitation. Although the initial lactate improved to 5.9 mmol/L after cardioversion, intermittent AF with rapid ventricular response resulted in worsening lactic acidosis and hypoxia, and he was intubated for airway protection. Shortly after intubation, the patient suffered a pulseless cardiac arrest but achieved return of spontaneous circulation (ROSC) within 3 minutes. Pericardiac arrest, an intra-aortic balloon pump (IABP) was inserted. Initially, therapies for his thyrotoxicosis included methimazole 20 mg every 6 hours and hydrocortisone 100 mg every 8 hours, to treat both AIT types 1 and 2. In addition, cholestyramine 4 grams every 6 hours was initiated to limit enterohepatic recycling of thyroid hormones. Although there may be overlap between AIT types 1 and 2, initial suspicion was for type 1, due to a possible undiagnosed toxic thyroid nodule with his physical exam findings of exophthalmos and lid lag. In the following day, after discussion with endocrinology, therapeutic plasma exchange (TPE) (PLEX) was initiated with a goal to achieve euthyroid thyroid function tests, until he would be hemodynamically stable for surgery. After one PLEX session, the TSH improved to 0.35 mIU/L, free T4 decreased to 1.6 ng/dL, and free T3 remained within range 2 pg/mL. Despite continued improvements in T3 and T4 after 2 more PLEX sessions, he remained in refractory shock in the CICU with cardiopulmonary collapse and refractory acidemia requiring continuous venovenous hemofiltration (CVVH). He subsequently arrested in the setting of monomorphic ventricular tachycardia (VT) and was not considered a candidate for venoarterial extracorporeal membrane oxygenation (VA ECMO) after discussion with the “SHOCK team” and expired.

## 4. Discussion

We report a case of attempted PLEX for the treatment of AIT in a patient with decompensated heart failure, who presented in cardiogenic shock. Our case demonstrates the importance of surveillance of thyroid function tests (TFTs) preamiodarone initiation, with continued monitoring, and the risks of delayed diagnosis, due to lack of consistent outpatient follow-up. Upon admission to the CICU and stabilization, a multidisciplinary discussion between the cardiac intensive care team including clinical pharmacy and endocrinology occurred to discuss pharmacologic therapies for suspected AIT, including potential preparation for TPE. In patients presenting with thyroid storm with cardiovascular collapse, estimated mortality remains high at 20-50% in the absence of thyroidectomy [6]. Treatment of thyroid storm associated with amiodarone includes discontinuation of amiodarone, when feasible, and initiation of antithyroid medications such as methimazole dosed at 20 mg every 4 to 6 hours or propylthiouracil dosed at 450 to 600 mg daily. Thionamides inhibit synthesis of thyroid hormone production by blocking oxidation of iodine in the thyroid gland and further synthesis of T3 and T4. These therapies may be continued, if amiodarone cannot be discontinued, up to 6 to 18 months after amiodarone cessation. However, as both PTU and MMI are hepatically metabolized, liver function tests (LFTs) must be monitored while on therapy, especially when used in conjunction with amiodarone. Even though our patient was initiated on methimazole immediately, LFTs significantly increased, likely secondary to shock liver. Although a goiter was not evident, serum thyroglobulin was elevated in our patient, and so treatment with hydrocortisone was initiated at 100 mg intravenously every 8 hours, along with methimazole. Glucocorticoids are recommended for the treatment of type 2 AIT for the anti-inflammatory and membrane-stabilizing effects, along with decreasing peripheral T4 to T3 conversion. The use of saturated potassium iodine solution (SSKI) for AIT to minimize intrathyroidal iodine content remains controversial and should only be initiated if there is a poor response to antithyroid medications. Other rare, adjunctive therapies include lithium carbonate or cholestyramine, to inhibit thyroid hormone secretion and bind thyroid hormones, respectively [11]. Despite these therapies, definitive therapy most often entails a thyroidectomy, which may not be feasible in patients presenting in cardiogenic shock, such as our patient. Therefore, the use of PLEX has been reported, to clear amiodarone and thyroid hormones from circulation rapidly [12–17]. Notably, despite the growing body of evidence to support PLEX for suspected AIT, patient criteria, timing of initiation of PLEX, and contraindications or considerations to this therapy remain elusive, with conflicting data showing benefit [12–17]. Even in the absence of amiodarone as the culprit, guidelines for the optimal role for PLEX in thyroid storm are not well established and the decision to initiate this therapy must be individualized. Further, hemodynamic instability during PLEX, especially in those presenting with cardiac arrest, may limit utility of PLEX for most patients presenting with severe AIT. Amiodarone and its metabolites, which are highly bound to plasma proteins, as well as free T3 and T4 molecules, have been shown to be cleared by TPE [17]. Indication for TPE is typically based on thyroid storm resistant to medical management and often involves daily TPE or every other day until euthyroid state is noted on thyroid function test. Thyroid function test should be repeated 2-3 hours after a session of TPE is completed. Our patient's poor response with hemodynamic instability and acute liver injury led to the decision to use PLEX as a bridge to thyroidectomy, despite the recent PEA arrest and critical illness, similar to a case of a patient that presented with sustained monomorphic VT due to AIT [16]. However, despite three PLEX sessions and improvement in TFTs, our patient suffered another cardiac arrest which subsequently led to his demise. His persistent metabolic derangements and acidosis were likely the precipitating factors of his monomorphic VT. Catheter ablation was not pursued given the acuity of the patient's condition and the likely etiology of his arrhythmia.

In conclusion, this case highlights the robust rapidly deleterious demise of AIT, specifically in patients with decompensated heart failure. Treatment modalities for AIT include discontinuing amiodarone, introducing thionamides and glucocorticoids, and decreasing the adrenergic response with beta blockers, if necessary. The decision to PLEX or not to PLEX for AIT should be individualized, but considered early in patients presenting with arrhythmias, to rapidly remove iodine and excess thyroid hormones from circulation, prior to definitive therapy. Still, mortality associated with AIT in those presenting with cardiogenic shock remains high.

## Figures and Tables

**Table 1:** Thyroid function tests.

	Latest reference range and units	06/01/20	11/02/20	03/23/21	04/05/21	08/03/22	09/12/22
T3, free	2.00-4.40 pg/mL		3.18				
T4, free, direct	0.80-1.90 ng/dL	1.57	1.76		1.69		
T4, free, nondialysis	0.7-1.5 ng/dL					2.2 (H)	3.1 (H)
Thyroid peroxidase antibodies	<35 IU/mL	10					
Thyroid-stimulating hormone	0.270-4.200 *μ*IU/mL	3.910	2.230		3.77		
TSH, high sensitivity	0.4-4.1 mIU/L			2.31		<0.02 (L)	<0.02 (L)

(H): data is abnormally high; (L): data is abnormally low.

## Data Availability

Data can be made available upon request to the corresponding author.

## Abbreviations

AF: Atrial fibrillation
AIT: Amiodarone-induced thyrotoxicosis
CICU: Cardiac intensive care unit
ECG: Electrocardiogram
IABP: Intra-aortic balloon pump
ICD: Intracardiac defibrillator
MMI: Methimazole
PLEX: Plasmapheresis
PTU: Propylthiouracil
ROSC: Return of spontaneous circulation
SSKI: Saturated potassium iodine solution
TPE: Therapeutic plasma exchange
T4: Thyroxine
TBG: Thyroxine-binding globulin
T3: Triiodothyronine
TTE: Transthoracic echocardiogram
TPO: Thyroid peroxidase antibody
VT: Ventricular tachycardia.

## References

[1] Goldschlager N., Epstein A. E., Naccarelli G. V. (2007). A practical guide for clinicians who treat patients with amiodarone: 2007. *Heart Rhythm*.

[2] Roy D., Talajic M., Dorian P. (2000). Amiodarone to prevent recurrence of atrial fibrillation. *The New England Journal of Medicine*.

[3] Tsang W., Houlden R. L. (2009). Amiodarone-induced thyrotoxicosis: a review. *The Canadian Journal of Cardiology*.

[4] Wong R., Cheung W., Stockigt J. R., Topliss D. J. (2003). Heterogeneity of amiodarone-induced thyrotoxicosis: evaluation of colour-flow Doppler sonography in predicting therapeutic response. *Internal Medicine Journal*.

[5] Rao R. H., McCready V. R., Spathis G. S. (1986). Iodine kinetic studies during amiodarone treatment. *The Journal of Clinical Endocrinology and Metabolism*.

[6] Bartelena L., Brogioni S., Grasso L., Bogazzi F., Burelli A., Martino E. (1996). Treatment of amiodarone-induced thyrotoxicosis, a difficult challenge: results of a prospective study. *The Journal of Clinical Endocrinology and Metabolism*.

[7] Newnham H. H., Topliss D. J., Le Grand B. A., Chosich N., Harper R. W., Stockigt J. R. (1988). Amiodarone-induced hyperthyroidism: assessment of the predictive value of biochemical testing and response to combined therapy using propylthiouracil and potassium perchlorate. *Australian and New Zealand Journal of Medicine*.

[8] Iudica-Souza C., Burch H. B. (1999). Amiodarone-induced thyroid dysfunction. *The Endocrinologist*.

[9] Daniels G. H. (2001). Amiodarone-induced thyrotoxicosis. *The Journal of Clinical Endocrinology and Metabolism*.

[10] Cardenas G. A., Cabral J. M., Leslie C. A. (2003). Amiodarone induced thyrotoxicosis: diagnostic and therapeutic strategies. *Cleveland Clinic Journal of Medicine*.

[11] Dickstein G., Shechner C., Adawi F., Kaplan J., Baron E., Ish-Shalom S. (1997). Lithium treatment in amiodarone-induced thyrotoxicosis. *The American Journal of Medicine*.

[12] Simsir I. Y., Ozdemir M., Duman S., Erdogan M., Donmez A., Ozgen A. G. (2018). Therapeutic plasmapheresis in thyrotoxic patients. *Endocrine*.

[13] Diamond T. H., Rajagopal R., Ganda K., Manoharan A., Luk A. (2004). Plasmapheresis as a potential treatment option for amiodarone-induced thyrotoxicosis. *Internal Medicine Journal*.

[14] Miller A., Silver K. D. (2019). Thyroid storm with multiorgan failure treated with plasmapheresis. *Case Reports in Endocrinology*.

[15] Burch H. B., Wartofsky L. (1993). Life-threatening thyrotoxicosis. Thyroid storm. *Endocrinology and Metabolism Clinics of North America*.

[16] Upadhyaya V. D., Douedi S., Akula M., Chalasani K. K., Saybolt M. D., Hossain M. (2020). Amiodarone-induced thyroid storm causing sustained monomorphic ventricular tachycardia treated with plasmapheresis: a challenging clinical case. *Journal of Medical Cases*.

[17] Muller C., Perrin P., Faller B., Richter S., Chantrel F. (2011). Role of plasma exchange in the thyroid storm. *Therapeutic Apheresis and Dialysis*.

